# A study on the effect of host plants on Chinese gallnut morphogenesis

**DOI:** 10.1371/journal.pone.0283464

**Published:** 2023-03-22

**Authors:** Qin Lu, Hang Chen, Jinwen Zhang, Weiwei Wang, Yongzhong Cui, Juan Liu

**Affiliations:** 1 Research Institute of Highland Forest Science, Chinese Academy of Forestry, Kunming, China; 2 Nanjing Forestry University, Nanjing, China; 3 The Key Laboratory of Cultivating and Utilization of Resources Insects of State Forestry Administration, Kunming, China; Central University of Haryana School of Life Sciences, INDIA

## Abstract

Galls are products of the hyperplasia of host plant structures induced by gall-inducing organisms and have been considered as an extended phenotype of the inducers. There is little evidence regarding the effect of host plants on gall morphology. We hypothesised that the morphology and developmental pattern of galls are different because of the different location of their stimulation, even though two kinds of inducers are close relatives. We observed that horned galls and their leaflets of their host plant, *Rhus chinensis* required a longer rapid growth stage than fusiform galls and *Rhus potaninii* leaflets. The distribution of trichomes showed positional dependence. Molecular analysis showed that in the fusiform gall, the target genes that regulate the plastochron of leaflets and serration development were hardly expressed, and *CUP-SHAPED COTYLEDON-2* may be a key gene that regulates the formation of the horns. In summary, horned and fusiform galls showed a developmental pattern similar to those of their host plant leaflets. We suggest that the inducing site is important in the morphology and development of galls.

## Introduction

Galls are products of cell hyperplasia induced by galling organisms on host plants. More than 15,000 species can induce gall formation, and the major inducers are mites, fungi, and insects [1]. Insects produce diverse and complex galls [2]. Galls can be induced on leaves, stems, branches, and roots, depending on the lifestyle of the inducers [3]. After settling in the host plant, gall-inducing organisms can change plant development through complex interactions with the host plant. A common change triggered by inducers is alterations in the cell cycle of the host plant, leading to an increase in the volume and quantity of cells (hyperplasia) [4–7]. There are some theories on how inducers control the host plant. For example, 1) Davey found that gall development in *Agrobacterium* can be induced and controlled by injecting bacterial DNA into the plant genome [8]. 2) Insects induce complex galls by changing the phytohormones of the host plant. 3) Through high-throughput sequencing, more interactive effectors were discovered, most of them were proteins, and these effector proteins come from salivary or poison gland of the inducer [9, 10]. However, the function of effector proteins has not been further verified. So, the mechanism of the gall formation remains unclear [11].

Galls induced by fungi and insects have similar biological functions. Galls provide a unique habitat to protect parasites from natural enemies and protect larva against actual body contact with rain, ice, snow, and sunlight [12, 13]. Some kinds of parasites feed on the inner wall of galls and exchange nutrients with the host plant via the wall, thus the gall is also the feeding place of these inducer [14, 15]. In a study in which *Lantana camara* L. (Verbenaceae) was fed by *Aceria lantanae* (Acarina: Eriophyidae) and *Schismatodiplosis lantanae* (Diptera: Cecidomyiidae), the resulting *A*. *lantanae* galls were uni- or multi-chambered and contained several mites, caused by several leaf folds, and consisted of hyperplasic epidermis and parenchyma. However, *S*. *lantanae* induced uni-chambered pouch galls inhabited by one larva or pupa, and the galls consisted of predominantly hypertrophied spongy parenchyma [13]. Therefore, the inducers play a key role in gall formation and control the galls to serve themselves, which is why galls have been considered as an extended phenotype of parasites [16, 17]. Galls can also be used as the basis for classifying inducers [5]. In summary, some reports suggest that the biological characteristics of galls are primarily determined by the inducers.

In other studies, gall formation was found to be affected by different vertical strata of the tree canopy and variations in leaf traits [18, 19]. Gall abundance on *Mangifera indica* leaves was found to be higher in the upper than the lower zone of the plant canopy, which on its turn was higher than in the inner zone [20]. In a type of aphidic gall, the number/size of the gall is different when a kind of aphid feeding on the different site of the same host [21]. In addition to size and number, the host plant also affects the morphogenesis of galls. The concentration of antioxidants in galls induced by *Ditylenchus gallaeformans* (Tylenchida: Anguinidae) on *Miconia ibaguensis* (Myrtales: Melastomataceae) was determined by both the stimulation of the inducer and the intrinsic physiological features of the host plant species [22]. “The insect galls are in a sense new plant organs because it is the plant that produces the gall in response to a specific stimulus provided by the invading insect” [23], so besides the inducer, the role of plants in gall morphogenesis is worth to studying.

The seration on the leaves are regulated by the *CUP-SHAPED COTYLEDON* (*CUC*) family, *ERECTA*, and *SQUAMOSA promoter binding protein* (*SPB*) [24, 25]. The *CUC* family of proteins is involved in the establishment and maintenance of organ boundaries [26]. In the *CUC* mutant of *Arabidopsis thaliana*, cotyledons were found to be fused along the edges of their sides, resulting in a cup-shape [27]. The *CUC* family regulates leaf serration, as evidenced by the fact that inactivation of *CUC-3* leads to partial suppression of serrations on the leaves of *A*. *thaliana* [28]. Furthermore, Kawamura et al. identified that *CUC-2* promotes the outgrowth of teeth in leaves through the regulation of auxin levels, and the level of auxin is higher in serrated leaves than round leaves [29]. In addition to maintaining boundaries, the *CUC* family also affects the initiation of the shoot apical meristem [30, 31]. *ERECTA*, a receptor-like kinase isolated from *Arabidopsis* LA-0 (Landsberg), can cooperate with a variety of genes to control the morphology of plant organs, including leaf morphology and stomatal development [32, 33]. Wild *A*. *thaliana* possesses oval leaves and long petioles, whereas the *ERECTA*-mutant possesses round leaves and a short petiole. This is because *ERECTA* regulates the quantity and volume of cells by maintaining a negative correlation between the number of epidermal cells and size of the cells and a positive correlation between the volume and number of rosette leaves [34]. *ERECTA* can also influence leaf shape by regulating the transport of auxin during leaf initiation; auxin accumulates in the L1 layer of the meristem and cannot flow into the hypocotyl vasculature in *ERECTA*-deficient plants, resulting rounder leaves in the mutant *A*. *thaliana* than in the wild type [35]. The *SPB* family also plays a role in plant development by regulating inflorescence, flowering time, juvenile-to-adult vegetative transition, and the vegetative-to-reproductive transition of the leaves (plastochron). The *SPB* family shows high expression when the leaves have a long plastochron, which indirectly affects yield [36–38]. Additionally, the *SPB* family regulates the rosette leaves. When researchers overexpressed microRNA (miR156) using a virus-based miRNA suppression system, miR156 repressed a group of *SPB* transcription factors (lower expression level of *SPB*), resulting in the absence of serration at the leaf edges [39].

*Kaburagia rhusicola* (Hemiptera: Pemphigidae) and *Schlechtendalia chinensis* (Hemiptera: Pemphigidae) are obligatory parasites that produce fusiform and horned galls on *Rhus potaninii* and *Rhus chinensis*, respectively [40]. Horned and fusiform galls are called Chinese gallnuts and are important commercial crops in forestry [41]. Fusiform and horned galls have certain common traits, such as specific reticulate schizogenous ducts associated with the vascular bundles, that fill the inner wall, and expanded xylem in the stalk that serves as a strong support and facilitates the exchange of nutrients [42]. As mentioned above, some studies have shown that the host plant can affect the anatomy and metabolism of galls [20–23]. In this study, we tested two galls produced by two galling aphids, and hypothesized that the morphology and developmental pattern differ because of their different stimulation sites.

## Materials and methods

### Developmental parameters

Leaflets of *R*. *potaninii* and *R*. *chinensis*, and fusiform and horned galls at different stages were collected in Wufeng County, Hubei Province, China. We collected the two kinds of galls and their host plant leaflets when they were 0.3 and 0.5 cm in length at the initial stage when they stopped growing and bursting, respectively. The dates and definitions of these stages are presented in Table 1. Leaflets were collected once a week, and galls were collected every 10 days because of their unique growth cycle. To analyse the developmental patterns efficiently, we divided the life cycle of the samples into five stages based on fold changes of weight, volume, length, width, wall thickness, and superficial area of leaflets, which were calculated based on parameters of the last collection/ parameters of the previous collection (every parameter underwent a fold change). When the fold change was more than double, the previous data were regarded as the beginning of the rapid growth stage, and when the fold change was less than twice, the later data were regarded as the end of the rapid growth stage. The first stage was the date we collected the first samples, the second stage was the beginning of fast growth, the third stage was the middle of rapid growth, the fourth stage was the end of rapid growth, and the fifth stage was the last time samples were collected (Table 1).

**Table 1:** Sample collection date and definitions of developmental stages.

*R*. *potaninii*		*R*. *chinensis*		Fusiform gall		Horned gall	
Date	Stage	Date	Stage	Date	Stage	Date	Stage
21 May	first	21 May	first	20 Apr	first	26 May	first
28 May		28 May		1 May		5 Jun	
5 Jun	second	5 Jun	second	11 May		15 Jun	second
12 Jun	third	12 Jun	third	21 May	second	25 Jun	
19 Jun		19 Jun	fourth	31 May	third	5 Jul	
26 Jun	fourth	26 Jun		10 Jun		15 Jul	
3 Jul		3 Jul		20 Jun	fourth	25 Jul	
10 Jul		10 Jul		30 Jun		05 Aug	third
16 Jul	fifth	16 Jul	fifth	10 Jul		15 Aug	
				20 Jul		25 Aug	fourth
				30 Jul	fifth	05 Sep	
						15 Sep	fifth

During collection, 30 *R*. *pontaninii* and 30 *R*. *chinensis* which had similar height, health, and place of growth were chosen, one gall and one leaflet were collected from a tree each time, and all 30 samples were used to test the developmental parameters (biological replicates). The length and width of samples and thickness of the gall wall were measured using a Vernier caliper. The volume of all samples was measured using the displacement method (samples were sunk in a measuring cylinder filled with water, and the increased volume was calculated), and the superficial area of leaflets was measured using an intelligent leaf area measuring instrument (LI-3000C, Palo Alto, CA, USA). The weight was measured using a microbalance (OLABO, Shanghai, China).

### Surface of samples and trichome count

Fresh samples were fixed in 4% glutaraldehyde for 2 hours, dehydrated using a series of ethyl alcohol concentrations (30% for 1 hour, 50% for 1 hour, 70% for 40 min, 80% for 30 min, 90% for 15 min, and 100% for 5 min). The dehydrated tissues were air-dried and sprayed with gold. The treated samples were observed using a scanning electron microscope (Tabletop Microscope 3000, Tokyo, Japan). The number of trichomes was simultaneously calculated.

### Tissue anatomy

The tissues from the first, third, and fifth stages were fixed in FAA solution, cut into 2–3 mm pieces, and dehydrated in an ethanol series (70% ethyl alcohol for 30 min, 80% for 20 min, 90% for 15 min, 95% for 10 min, and 100% for 5 min). Then the ethyl alcohol was replaced with xylene and paraffin. The gall samples were embedded in paraffin, 5‐μm‐thick sections were prepared using a rotary microtome (Leica RM2126RT, Solms, Hesse‐Darmstadt, Germany), and the leaflets were cut into 4‐μm‐thick sections. The sections were de-paraffinized and stained with safranin and Fast Green after parching. Then 20 sections of each tissue type were measured for cell size and cell layer quantity.

### Data analysis

Since our aim was to study the developmental pattern of the samples, we used the fold changes of variable, instead of real parameters. Fold change were calculated by dividing the parameters of the next collection by the parameters of the current collection. Significant differences and regression analysis were analyzed by SPSS 20.00, significant differences were tested based on compared means, with one-way ANOVA using L-SD and P < 0.05. Linear regression was used for regression analysis and dependent variable was the number of the serration and the horn, independent variable was the gene expression level, P < 0.05.

### qRT-PCR

The sequences of the target genes were selected based on transcriptomes generated from previous studies (transcriptomes were submitted to the National Centre for Biotechnology Information Sequence Read Archive database, and can be found at the following sites: https://www.ncbi.nlm.nih.gov/bioproject/PRJNA631065 and https://www.ncbi.nlm.nih.gov/bioproject/PRJNA662477) [43, 44]. Specific primers were designed using Primer-primer 6.0, and the relative quantification method was used, with the first stage leaf samples as the control and tubulin as the reference gene. The primers used are listed in Table of the supporting information (S1 Table). The samples at different stages were submerged in liquid nitrogen for 10 min and stored at -80°C for later use. Total RNA was extracted using the TaKaRa MiniBEST Plant RNA Extraction Kit (TaKaRa, Dalian, China), according to the manufacturer’s instructions. First-strand cDNA was generated using the PrimeScript^™^ RT Reagent Kit with gDNA Eraser (Perfect Real Time) (TaKaRa, Dalian, China). A reaction mixture of 25 μL was prepared, which contained 1 μL sense primer (0.5 μM), 1 μL anti-sense primer (0.5 μM), 1 μL template (150 ng/μL), and 22 μL master mix in QuantStudio 7 Flex (Thermo Fisher, Waltham, MA, USA). The reaction procedure consisted of 40 cycles of denaturation at 94°C for 1 min, annealing at 53°C for 1 min, and extension at 72°C for 30 s. Expression levels were calculated according to CT values [34]. Three biological and three technical replicates were used. Regression analysis was performed by SPSS 20.0, and the dependent variable was the number of horns on horned galls or the number of serrations on *R*. *chinensis* leaflets. The expression level of each gene was used as an independent variable; R^2^ > 0.5.

No permit is needed in this study because of the host plant and the lab is public, and all software for analysis is open source.

## Results

### Morphology of galls and their host plants

**Fig 1:**
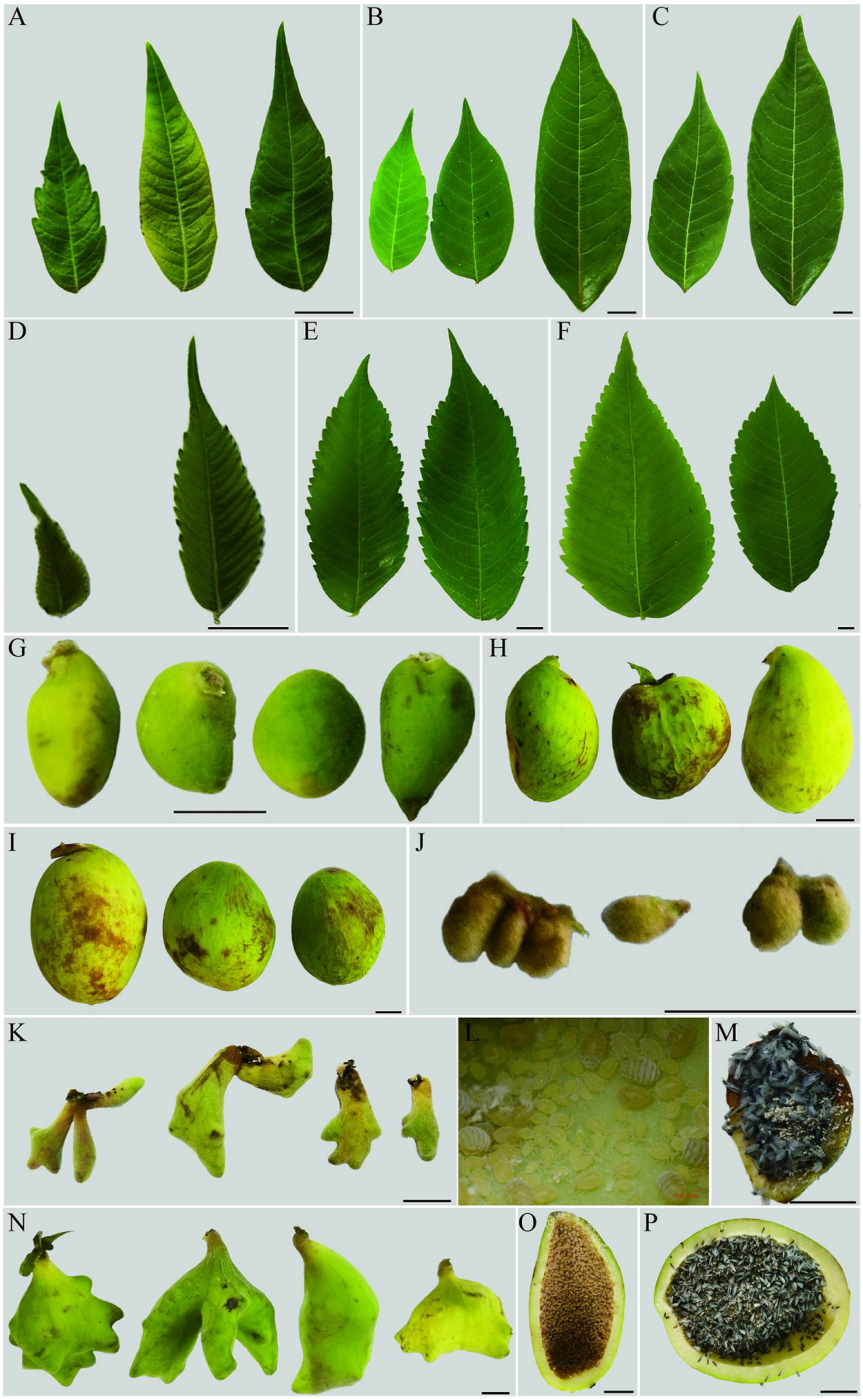
Morphology of *R*. *potaninii* and *R*. *chinensis* leaflets, horned and fusiform galls at different stages. (A) *R*. *potaninii* leaflets at initial stage. (B) The *R*. *potaninii* leaflets at third stage. (C) *R*. *potaninii* leaflets at fifth stage. (D) *R*. *chinensis* leaflets at initial stage. (E) *R*. *chinensis* leaflets at third stage. (F) *R*. *chinensis* leaflets at fifth stage. (G) Fusiform galls at initial stage. (H) Fusiform galls at third stage. (I) Fusiform galls at fifth stage. (J) Horned galls at initial stage. (K) Horned galls at third stage. (L) *S*. *chinensis* within horned gall at third stage. (M) *S*. *chinensis* within horned gall at fifth stage. (N) Horned galls at fifth stage. (O) *K*. *rhusicola* within fusiform gall at third stage. (P) *K*. *rhusicola* within fusiform gall at fifth stage. (A–K, M-P) scale bars = 1 cm; (L) scale bar = 200 μm.

The morphology of the two kinds of leaflets used in this study was remarkably distinctive: The *R*. *potaninii* leaflets were oval, had few serrations, and no newly formed serrations with leaf growth, and the leaflets were slightly leathery at a later stage (Fig 1A–1C). We found many serrations on *R*. *chinensis* leaflets, which could be observed from the initial to the later stages (Fig 1D–1F).

*K*. *rhusicola* and *S*. *chinensis* induce fusiform and horned galls on *R*. *potaninii* leaves and *R*. *chinensis* rachis wings, respectively. At the initial developmental stage (first stage), the fusiform and horned galls were round (Fig 1G and 1J), but their appearances started to show differences with growth. At the middle stage (third stage) of gall development, the fusiform galls remained oval in shape (Fig 1H), while extreme variation occurred in the horned galls. Some angular bulges formed on the horned galls (Fig 1K), and these bulges enlarged with gall development, which accounted for a third of the gall’s length (Fig 1N). Moreover, small horns occurred on the large horns (Fig 1N). The morphology of the fusiform galls was not significantly different from the initial to the late stages, except for an increase in volume (Fig 1G–1I). The morphology of the aphids inside the galls also changed; wingless aphids (Fig 1M and 1P) changed to winged aphids (Fig 1L and 1O).

### Developmental pattern of galls and their host plant leaflets

**Fig 2:**
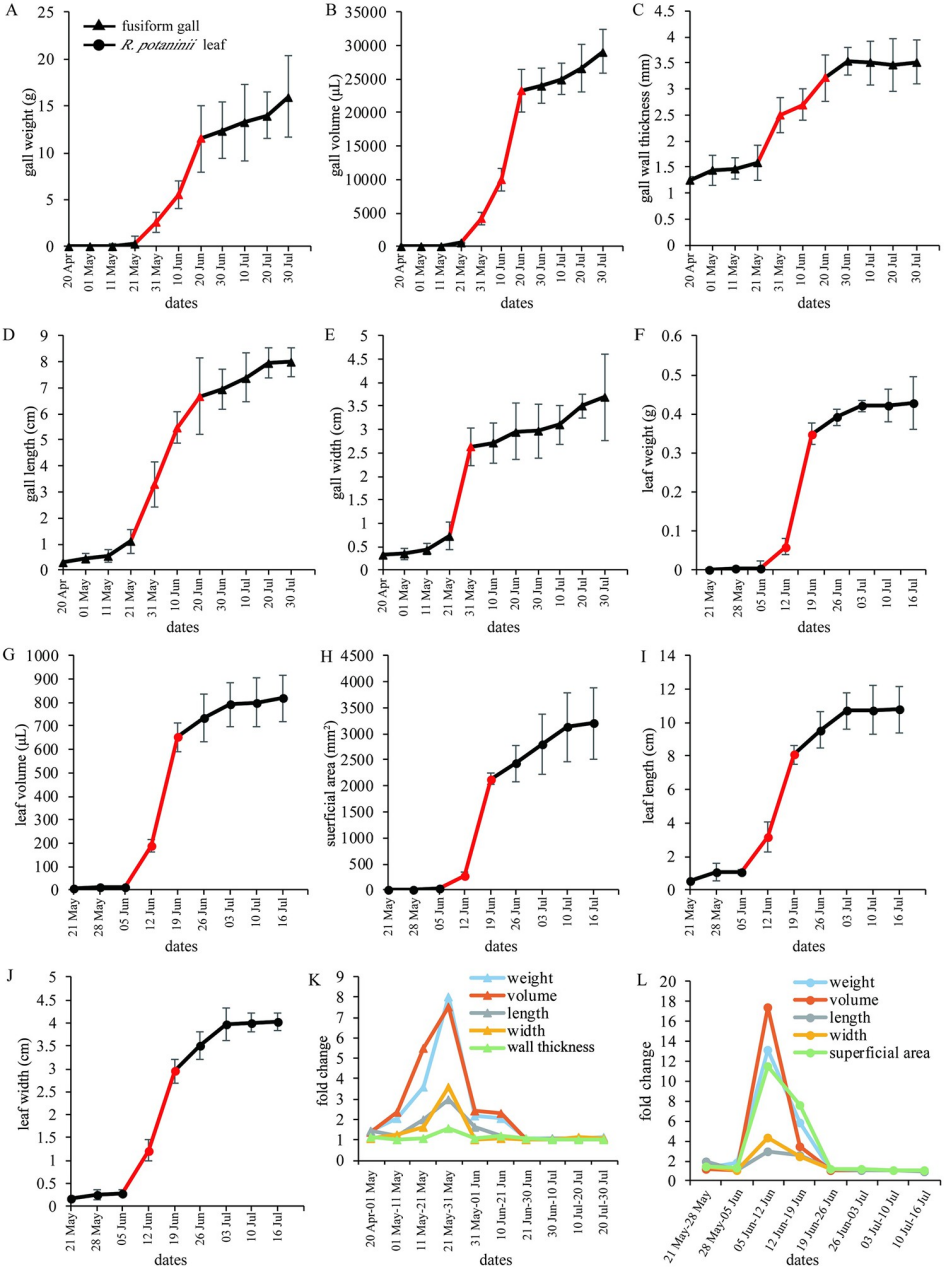
Developmental parameters of fusiform galls and *R*. *potaninii* leaflets. (A-E) Weight, volume, thickness, length, and width of fusiform galls during developmental stage, respectively. (F-J) Weight, volume, superficial area, length, and width of *R*. *potaninii* leaflets during developmental stage, respectively. (K-L) Fold change of developmental parameters of fusiform galls and *R*. *potaninii* leaflets. Growth curves indicate that development could be divided into initial, rapid growth, and slow growth stages. X axis of indicates the data of sample collection; red lines indicate rapid growth periods.

The weight, volume, length, width, thickness (gall wall), and superficial (leaf) area of fusiform galls and leaflets of *R*. *potaninii* were calculated. The growth cycle was 102 days (20 April to 30 July) for fusiform galls and approximately 47 days for *R*. *potaninii* leaflets (31 May to 16 July). The developmental period can be divided into three stages: initial, rapid growth, and slow growth stages (late stage) (Fig 2). A period of marked growth was observed in both *R*. *potaninii* leaflets and fusiform galls. Fusiform galls grew rapidly from 21 May to 20 June (30 days), and *R*. *potaninii* leaflets grew rapidly from 5 to 19 June (14 days) (Fig 2A–2J). Thereafter, fusiform galls and *R*. *potaninii* leaflets continued to grow, but at a much lower rate.

The fold changes (parameters of the next collection/ parameters of current collection) clearly indicated the developmental pattern of fusiform galls and *R*. *potaninii* leaflets: they grew slowly at the initial and later stages of development, and the fold change in these two periods was less than double. During the rapid growth period of fusiform galls, growth occurred fastest from 21 to 31 May, and the galls grew in weight and volume by eight times. A similar growth rate was observed in *R*. *potaninii* leaflets, but on a larger scale. The developmental peak occurred from 5 to 12 June, and the weight and volume increased by approximately 18 times (Fig 2K and 2L). In general, during the rapid growth stage (21 May to 30 June for fusiform galls and 5 to 19 June for *R*. *potaninii* leaflets), the length and width of fusiform galls increased by an average of five times, and the weight and volume increased by 39 and 43 times, respectively. The length and width of *R*. *potaninii* leaflets increased by an average of nine times, and the weight and volume increased by an average of 74 and 61 times, respectively.

**Fig 3:**
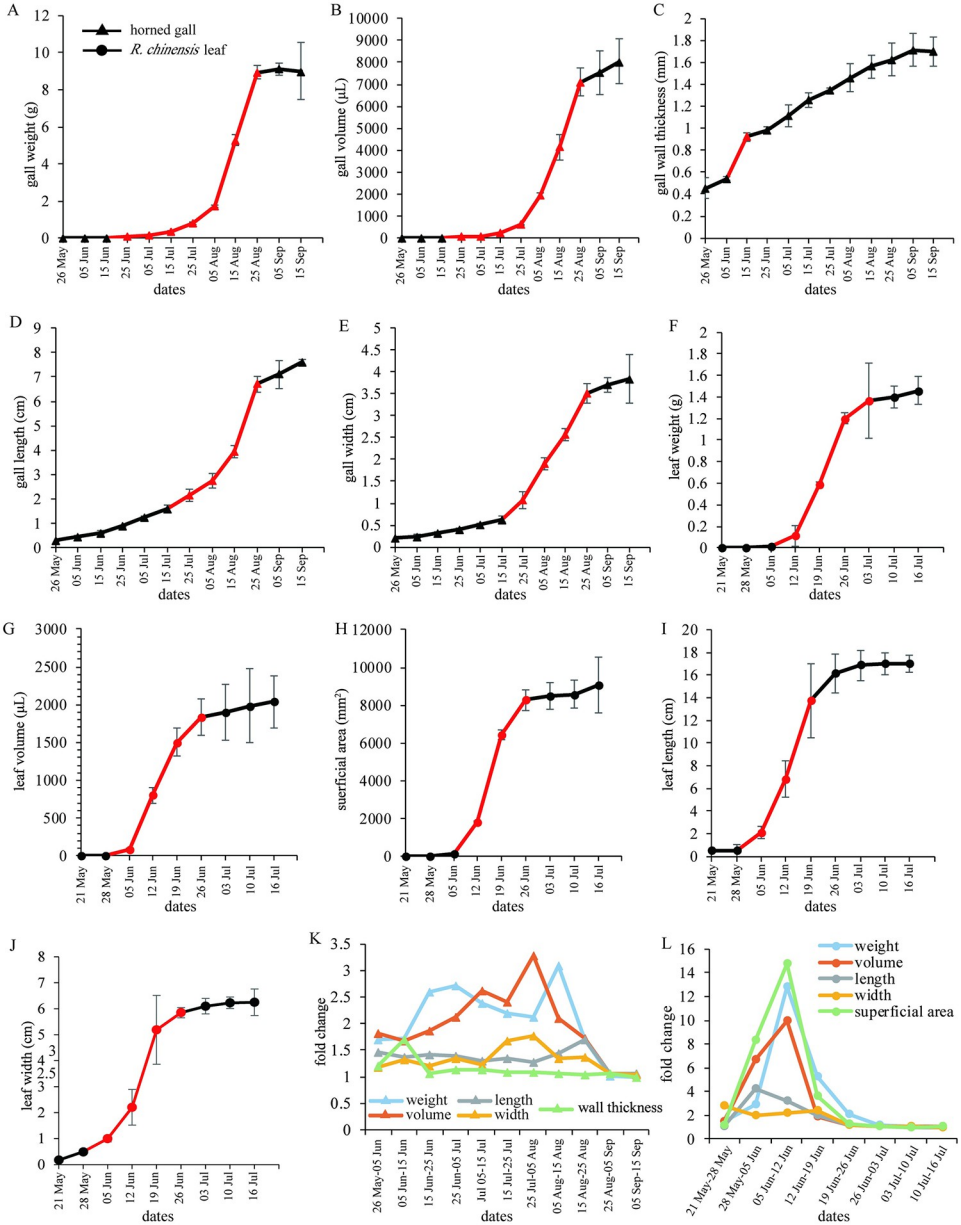
Developmental parameters of horned galls and *R*. *chinensis* leaflets. (A-E) Weight, volume, thickness, length, and width of horned galls during whole developmental stage, respectively. (F-J) Weight, volume, superficial area, length, and width of *R*. *chinensis* leaflets during whole developmental stage, respectively. (K-L) Fold change of developmental parameters of horned galls and *R*. *chinensis* leaflets. Growth curves indicate that development could be divided into initial, rapid growth, and slow growth stages. X axis indicates data of sample collection; red lines indicate rapid growth periods.

The growth cycle of horned galls was 112 days (26 May to 15 September), and for *R*. *chinensis* leaflets was approximately 47 days (31 May to 16 July). The developmental period can also be divided into initial, rapid growth, and slow growth (late) stage (Fig 3). The horned gall weight and volume had a longer rapid growth stage from 15 June to 25 August (72 days) compared to fusiform galls, and the length and width increased rapidly from 15 July to 25 August (45 days) (Fig 3A–3E). The rapid growth stage of *R*. *chinensis* lasted for 21 days (5 to 26 June). (Fig 3F–3J).

The fold changes suggested that horned galls maintained a long rapid growth stage; however, the rate of increase was much lower than that of fusiform galls, with a peak of weight growth from 5 to 15 August (2.7 times) and volume growth from 25 July to 5 August (3.3 times) (Fig 3K). The growth peak of *R*. *chinensis* leaflets was from 5 to 12 June, and the fold change of the superficial area was 14.7 times in this period (Fig 3L).

### Appendages of samples and correlation analysis

**Fig 4:**
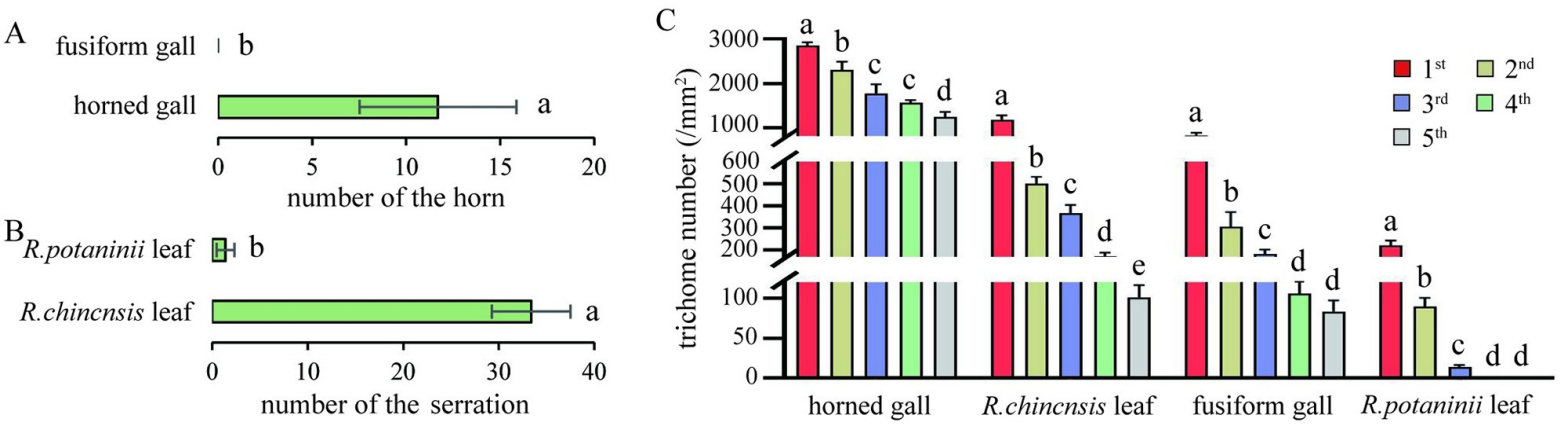
The number of horn, serration and trichome. A: The horn number of fusiform galls and horned galls. B: The serration number of *R*. *potaninii* leaflet and *R*. *chinensis* leaflet. C: The number of trichomes at different stages in the samples. P < 0.05.

We did not observe horns on the fusiform galls but we found a certain number of horns (11.69 ± 4.18) on the horned galls (Fig 4A). The *R*. *chinensis* leaflets had the most serrations (33.38 ± 4.12) and the *R*. *potaninii* leaflets had only a few serrations (1.38 ± 0.92) (Fig 4B).

The number of trichomes was also calculated at the different stages in the four samples: the horned galls had the most tomentum during all stages, with more than 2,000 trichomes/mm^2^ from the first to second stage and more than 1,000 trichomes/mm^2^ from the third to fifth stage. This was followed by their host leaflets (*R*. *chinensis* leaflets), with 1,177.87 trichomes/mm^2^ at the first stage and 500.87, 366.01, 172.83, and 101.2 trichomes/mm^2^ at the second, third, fourth, and fifth stages, respectively. The fusiform galls also possessed trichomes; but the amount of tomentum was smaller than that in horned galls during the whole life cycle, with 824.04, 305.87, 182.07, 105.68, and 83.24 trichomes/mm^2^ on the surface of fusiform galls at the first, second, third, fourth, and fifth stages, respectively. The *R*. *potaninii* leaflets had the least tomentum, with 222.28, 89.55, 13.83, and 0.08 trichomes/mm^2^ on their surface at the first, second, third, and fourth stages, respectively, and no tomentum at the fifth stage. The overall trend of tomentum distribution among all samples were a gradual decrease with growth (Fig 4C).

The images reflect the distribution pattern more intuitively, showing horned galls and *R*. *chinensis* leaflets (abaxial side) covered with dense tomentum at the initial stage (S1G and S1J Fig), a significant decrease in trichomes on *R*. *chinensis* leaflets at the third stage, stomata and glandular trichomes on the abaxial side, and less tomentum at the fifth stage (S1H and S1I Fig). At the third stage of horned gall development, the surface was still covered with tomentum, and stomata were observed until the fifth stage (S1K and S1L Fig). Fusiform galls and leaflets of *R*. *potaninii* showed a different pattern, with fewer trichomes compared to *R*. *chinensis*, and stomata and glandular trichomes were observed at the initial stage, which decreased significantly from the third stage, with a clear surface presentation of *R*. *potaninii* leaflets and fusiform galls (S1A–S1F Fig).

### Anatomical structure of galls and their host plant leaflets

**Fig 5:**
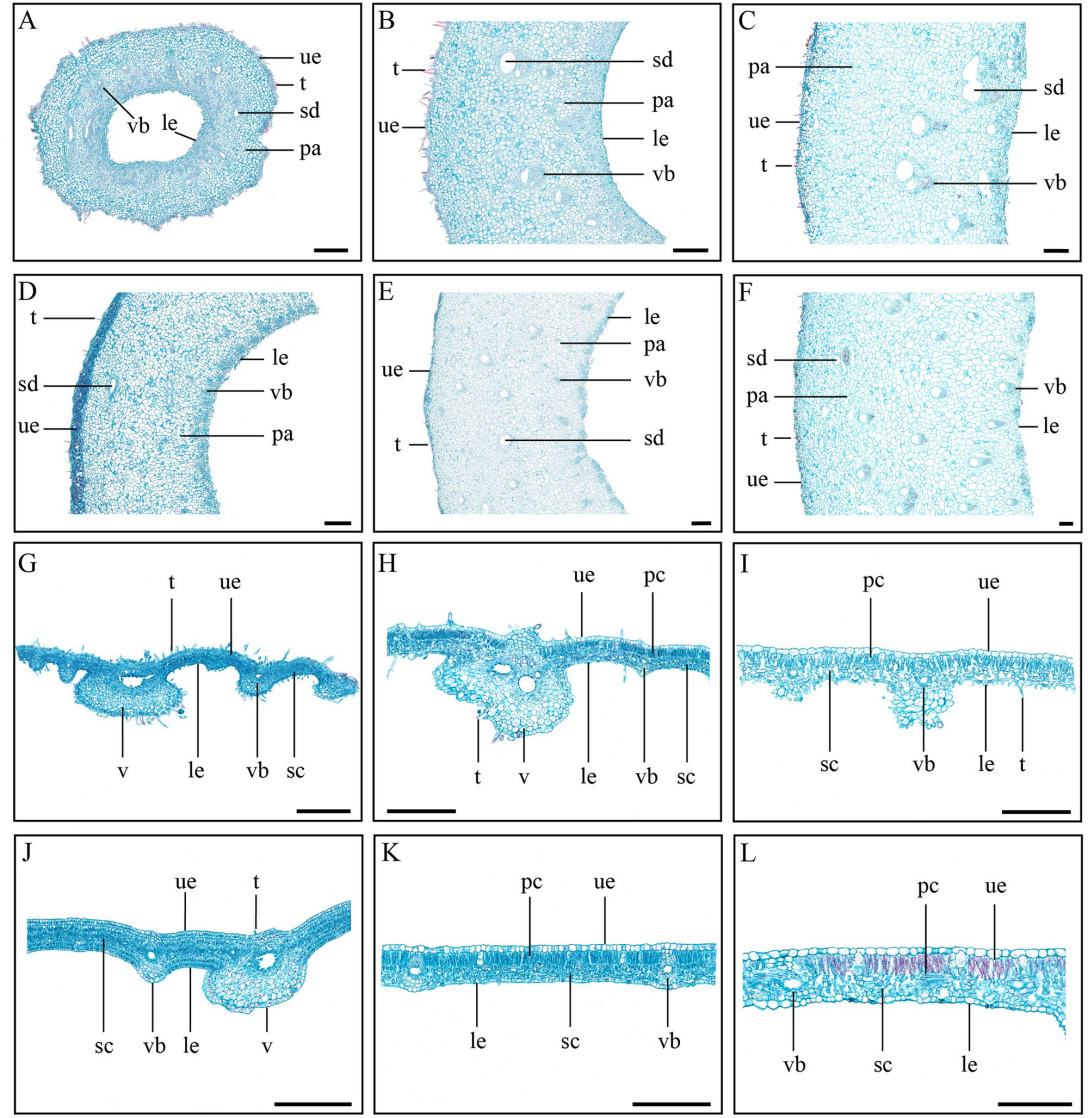
Histological profiles of galls and their host plant tissues. (A-C) Histological structure of horned gall at first, third, and fifth stage, respectively. (D-F) Histological structure of fusiform gall at first, third, and fifth stage, respectively. (G-I) Histological structure of *R*. *chinensis* leaflets at first, third, and fifth stage, respectively. (J-L) Histological structure of *R*. *potaninii* leaflets at first, third, and fifth stage, respectively. pa, parenchyma; sd, schizogenous duct; vb, vascular bundle; t, tomentum; pc, palisade cell; sc, spongy cell; le, lower epidermal cell; ue, upper epidermal cell; v, vein; Scale bars = 200 μm.

Samples from the first, third, and fifth stage- were examined using paraffin section. The histological features of horned and fusiform gall were similar. The wall was full of parenchyma with interspersed schizogenous ducts and vascular bundles. Epidermal cells were observed on the surfaces of the galls (Fig 5A–5F). The leaflets of *R*. *chinensis* and *R*. *potaninii* showed similar features. At the first stage, cells were closely arranged and had no organizational differentiation; palisade cells and upper epidermal cells appeared from the second stage onwards, and spongy cells were loosely arranged at the fifth stage. The galls possessed vascular bundles which also could be find in the leaflets (Fig 5G–5L).

**Fig 6:**
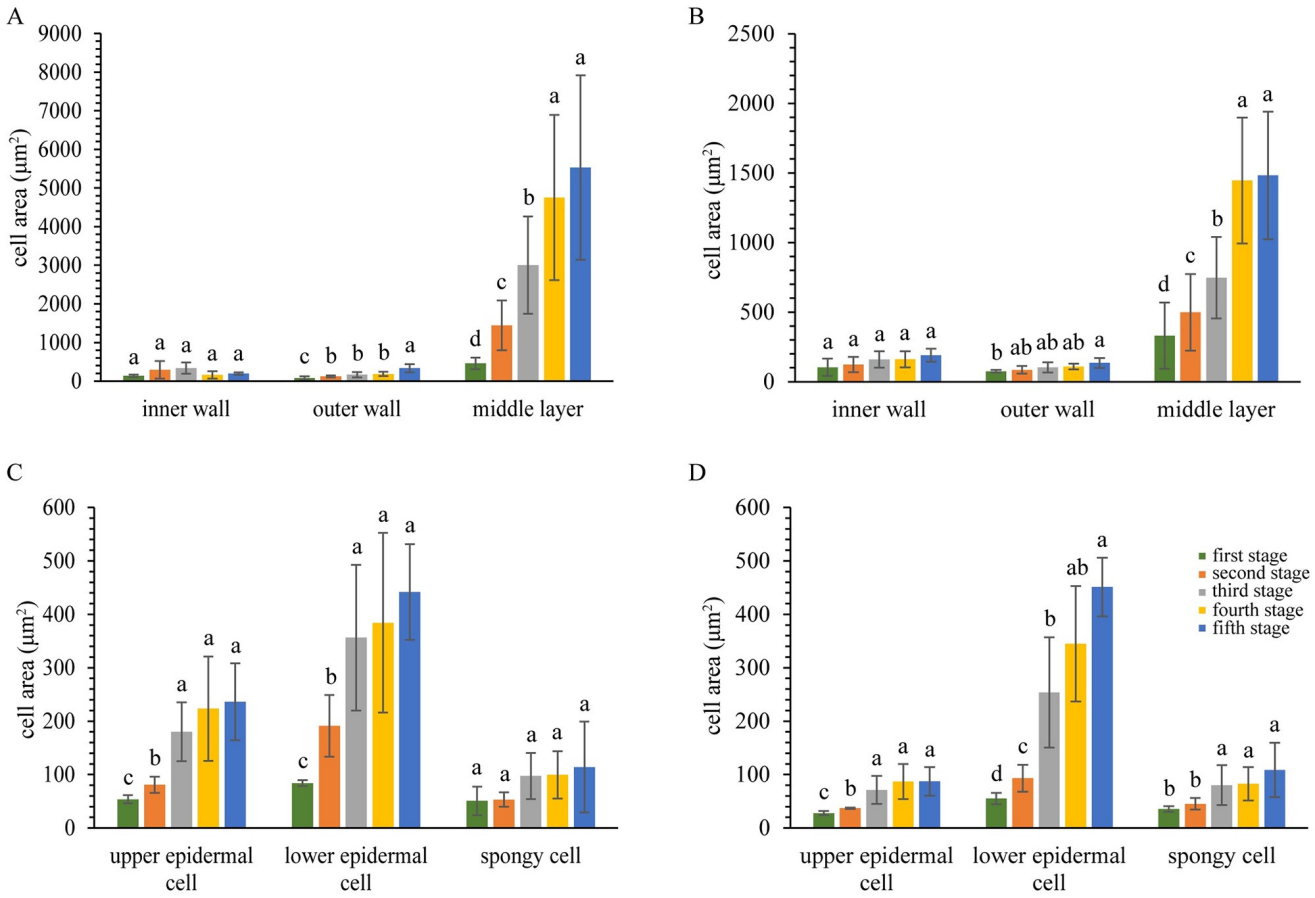
Cell area of fusiform and horned galls, and *R*. *potaninii* and *R*. *chinensis* leaves. (A) Cell area of fusiform galls. (B) Cell area of horned galls. (C) Cell area of *R*. *potaninii* leaves. (D) Cell area of *R*. *chinensis* leaves. P < 0.05. Lowercase letters indicate significant differences among five stages of each kind of cell, with average values decreasing from a to c, and no significant difference if they have the same letter.

Similar quantities of cell layer were found in *R*. *chinensis* and *R*. *potaninii* leaflet; they made of a upper epidermal cell layer, a lower epidermal cell layer, a palisade cell layer and four to five spongy cell layers. The quantity of each cell layer remained (Table 2), but the epidermal cell area became larger with leaflets growth. The upper epidermal cell area on *R*. *potaninii* leaflet was always larger than on *R*. *chinensis* leaflet (Fig 6C and 6D).

**Table 2:** Number of cell layer among four kinds of tissue at different stages.

		Horned gall	Fusiform gall	*R*. *chinensis* leaflets	*R*. *potaninii* leaflets
First stage	Total	31.67 ± 4.12c	40.43 ± 4.37c	7.00 ± 1.00a	6.50 ± 0.50a
	Outer wall/upper epidermal cells	1.00 ± 0.00	8.37 ± 3.96	1.00 ± 0.00	1.00 ± 0.00
	Inner wall/lower epidermal cells	8.03 ± 1.27	4.38 ± 1.05	1.00 ± 0.00	1.00 ± 0.00
Second stage	Total	35.85 ± 1.58c	48.25 ± 5.54b	6.20 ± 0.40a	6.75 ± 0.83a
	Outer wall/upper epidermal cells	1.00 ± 0.00	9.75 ± 2.28	1.00 ± 0.00	1.00 ± 0.00
	Inner wall/lower epidermal cells	7.54 ± 0.95	5.25 ± 1.48	1.00 ± 0.00	1.00 ± 0.00
Third stage	Total	46.25 ± 2.86b	55.2 ± 6.67b	6.25±0.43a	6.00 ± 1.00a
	Outer wall/upper epidermal cells	1.00 ± 0.00	4.33 ± 0.47	1.00 ± 0.00	1.00 ± 0.00
	Inner wall/lower epidermal cells	7.46 ± 1.48	2.75 ± 0.43	1.00 ± 0.00	1.00 ± 0.00
Fourth stage	Total	50.50 ± 3.20b	67.33 ± 3.30a	6.20± 0.40a	6.50 ± 0.50a
	Outer wall/upper epidermal cells	1.00 ± 0.00	3.54 ± 0.47	1.00 ± 0.00	1.00 ± 0.00
	Inner wall/lower epidermal cells	4.67 ± 0.47	1.50 ± 0.50	1.00 ± 0.00	1.00 ± 0.00
Fifth stage	Total	58.50 ± 4.61a	68.2 ± 5.64a	6.50 ± 0.50a	6.00 ± 0.00a
	Outer wall/upper epidermal cells	1.00 ± 0.00	1.33 ± 0.47	1.00 ± 0.00	1.00 ± 0.00
	Inner wall/lower epidermal cells	4.67 ± 0.47	3.20 ± 0.75	1.00 ± 0.00	1.00 ± 0.00

Cell layers of horned and fusiform gall were divided into total, inner wall, and outer wall. Cell layers of two kinds of leaflets were divided into total, upper epidermal cells, and lower epidermal cells. P < 0.05. Lowercase letters indicate significant differences among five stages, with average values decreasing from a to c, and no significant difference if they have the same letter.

Larger fusiform gall always possessed more cell layers, and there were 40.43 ± 4.37 parenchymal cell layers at the first stage; the number of parenchymal cell layers showed no significant differences from the second to third stage, and showed 67.33 ± 3.30 at the fourth stage. The cell layer number increased with fusiform gall growth except fourth to fifth stage (Table 2). The cell layer quantity of horned galls showed a similar trend with fusiform galls; the layers increased with horned gall growth; horned galls always showed fewer cell layers than fusiform galls. An upper epidermal cell layer could be found throughout horned gall development, and there were 8.03 ± 1.27 parenchymal cell layers on the inner wall, which always lower than the middle layer at the first stage. The small parenchymal cell layers decreased with horned gall growth. Differ from horned galls, small parenchymal cells could be found on both the outer and inner wall of fusiform galls, and the small parenchymal cell layers decreased with fusiform gall growth as well (Table 2).

Besides the cell layer quantity, the parenchymal cell area on the two kinds of gall also showed large difference. The parenchymal cell area on the inner wall had no significant difference throughout the growth of horned and fusiform galls. In fusiform galls, the parenchymal cell area on the outer wall increased with the gall development and the increase occurred mainly at the first to second and fourth to fifth stage. The increased area on the middle layer showed a bigger scale, with the area of parenchymal cells was 5527.68 ± 2387.72 μm^2^ at the fifth stage (Fig 6A).

In horned galls, the parenchymal cell area on the outer wall increased slowly, but increased significantly on the middle wall with gall development, except fourth to fifth stage (Fig 6B). The parenchymal cell area on the middle layer of horned and fusiform galls differed slightly at the first stage, but a larger difference occurred from the second stage, when the parenchymal cell area of fusiform gall wall more than three times that of horned gall wall (Fig 6A and 6B).

### Quantitative real-time PCR (qRT-PCR) of serration development-related genes

*CUC-2*, *CUC-3*, two *ERECTA* genes (*ERECTA-1* and *ERECTA-2*), *SPB-13*, and *SPB-16* were detected in the two types of galls and their host leaflets. Tubulin was used as the reference gene for these two species.

**Fig 7:**
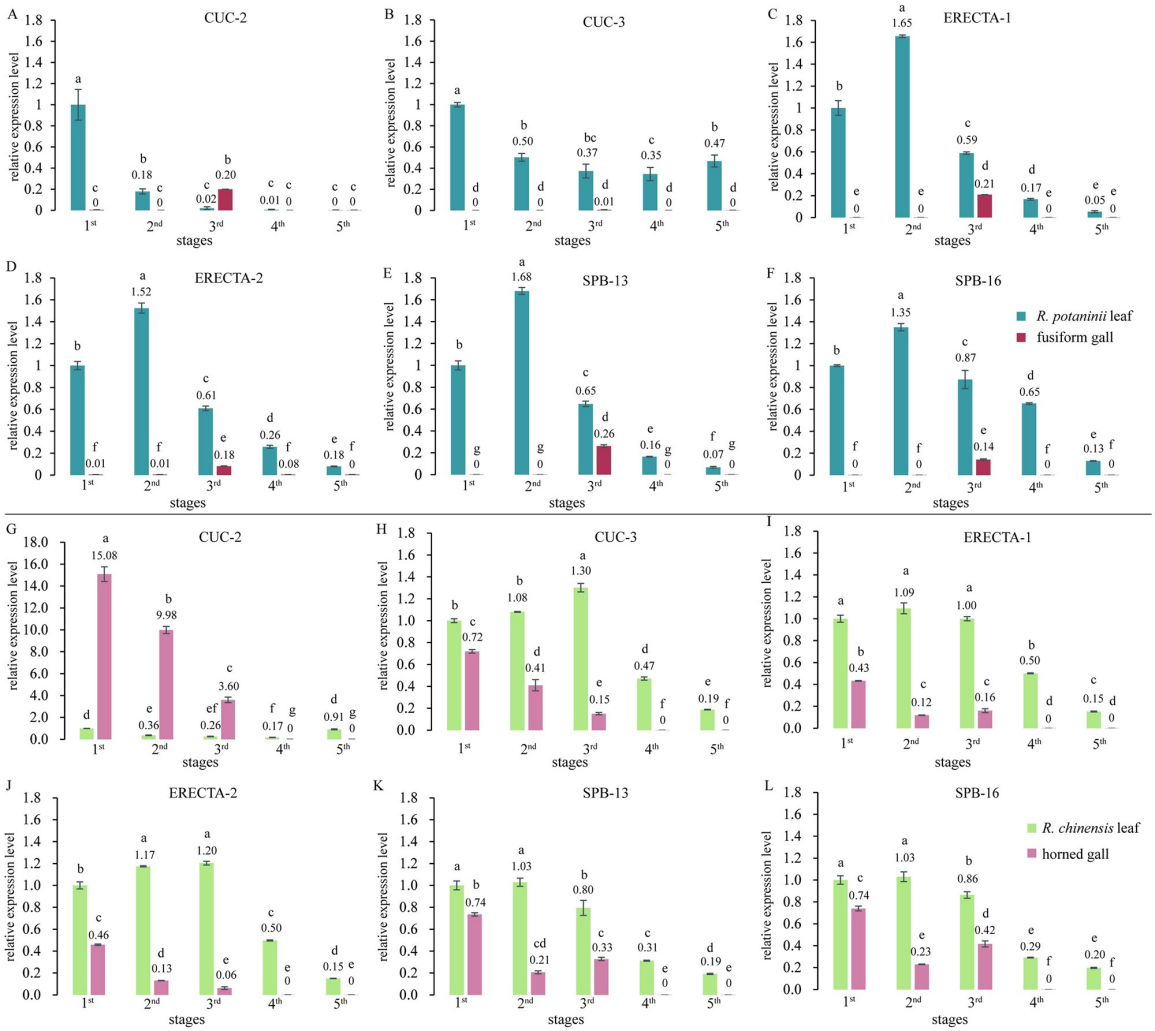
qRT-PCR analysis of genes regulating serration development at different stages. (A-F) Relative expression levels of candidate genes in *R*. *potaninii* leaflets and fusiform galls. (G-L) Relative expression levels of candidate genes in *R*. *chinensis* leaflets and horned galls. Gene expression levels were high in *R*. *potaninii* and *R*. *chinensis* leaflets and low in fusiform galls that did not possess horns; gene expression levels in *R*. *chinensis* leaflets and horned galls were high in first, second, and third stages. Lowercase letters indicate significant differences among five stages of each gene in gall and host plant, with average values decreasing from a to g, and no significant difference if they have the same letter. P < 0.05.

In *R*. *potaninii* leaf tissues, the expression levels of *CUC-2* and *CUC-3* was higher at the initial stage than at the other stages; however, *ERECTA-1*, *ERECTA-2*, *SPB-13*, and *SPB -16* showed expression peaks at the second stage. The expression levels decreased from the third development stage, and we detected no gene expression during the fifth stage, except for *CUC-3*. In t fusiform gall tissues, none of the genes were expressed during the life cycle, except in the third stage (Fig 7A–7F). In horned gall and its host leaflets, *CUC-2* showed a different expression pattern: high expression from the first to third stage, then decreasing significantly with gall development (Fig 7G). Genes were highly expressed in leaf tissues, and their expression decreased significantly from the fourth stage. In contrast to fusiform gall, all genes were expressed in horned gall tissues from the first to third stage, with expression levels more than one-half of those in leaflets at the first stage for most genes. No expression of *CUC-2*, *CUC-3*, *ERECTA-1*, *ERECTA-2*, *SPB-13* and *SPB-16* could not be detected at the fourth and fifth stage of horned gall development (Fig 7G–7L).

The regression analysis showed that *CUC-2* played a key role in the formation of horns on horned galls, followed by *SPB-16*. *ERECTA-2* seemed to have the opposite effect. *CUC-3*, *ERECTA-1*, and *SPB-13* had no significant effect on the formation of horns. The regulatory mechanism of serrations showed a similar pattern: *ERECTA-2* and *SPB-13* had no significant effect on the formation of serrations, and *CUC-2* played a key role, followed by *ERECTA-1*. *CUC-3* and *SPB-16* had an antagonistic effect (Table 3).

**Table 3:** Influence of each gene on morphogenesis.

	Standardized beta coefficients (horn)	Standardized beta coefficients (serrations)
*CUC-2*	1.64	3.667
*CUC-3*	-	-0.767
*ERECTA-1*	-	0.636
*ERECTA-2*	-2.901	-
*SPB-13*	-	-
*SPB-16*	1.530	-1.938

Positive numbers indicate that the gene has a positive effect on horn or serration formation; negative numbers indicate that the gene has a negative effect. Higher numbers indicate bigger impact. P < 0.05.

## Discussion

**Fig 8:**
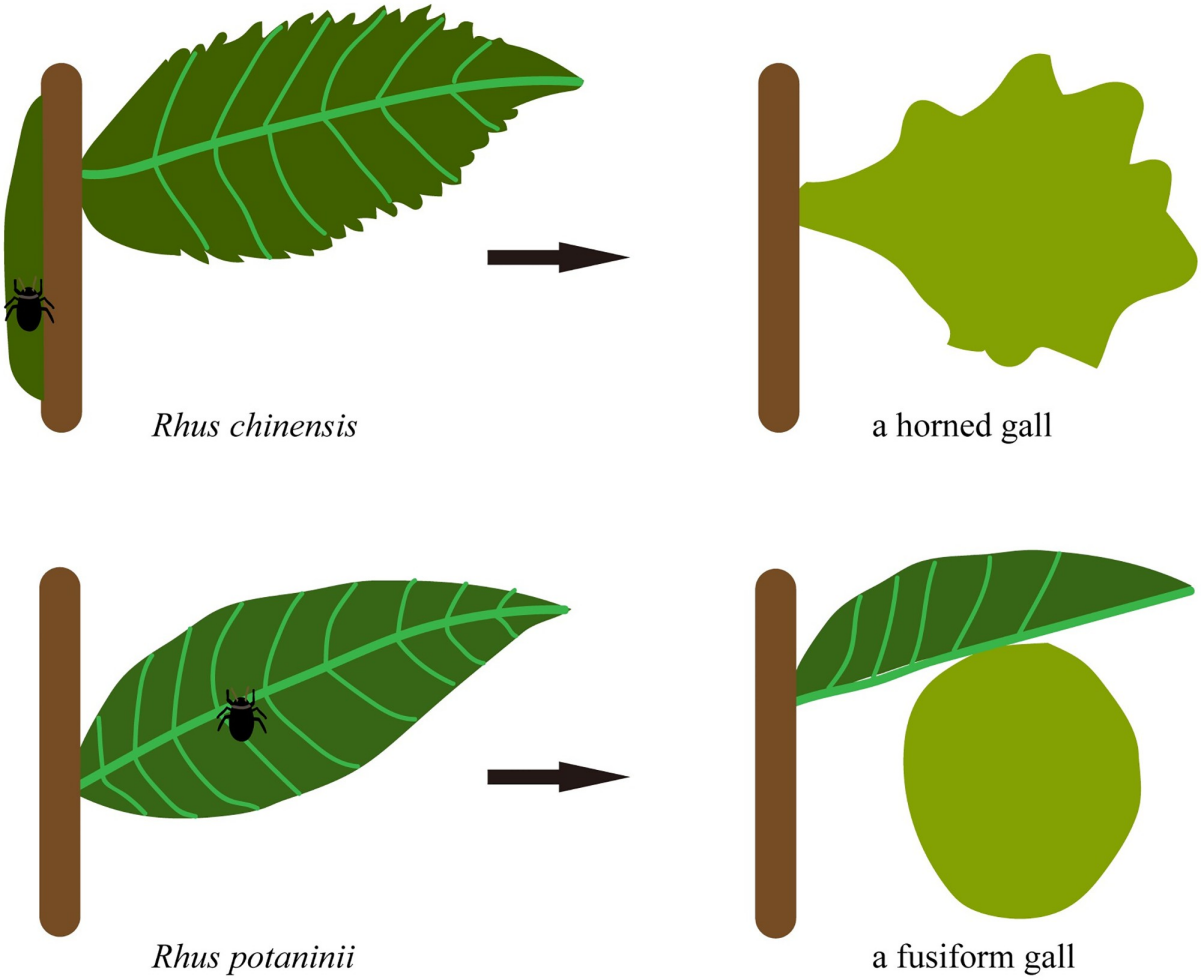
A model of gall formation. A round fusiform gall develops on the round *R*. *potaninii* leaflet, a horned gall develops on the serrated *R*. *potaninii* leaflet.

Galls are the outcomes of interaction between the galling organisms and their host plant, “the galls are usually species-specific structures, and their anatomy and metabolism are strongly related to the gall inducer species and to the host plant” [45]. Our study provides evidence on how the host plant affects the morphogenesis of galls based on tests of two closely relative aphids (Fig 8). We found that the number of horns on the galls was significantly positively correlated with the serrations on the leaflets of the host plant. Fusiform galls (no horns) located on *R*. *potaninii* leaflets had few serrations, and horned galls (11.69 ± 4.18 horns) grown on *R*. *chinensis* had the most serrations (33.38 ± 4.12). Genes that regulate serration development provide strong evidence. During the entire growth period of fusiform galls, we detected hardly any expression of the target genes, except in the third stage. Low *ERECTA* expression level was detected in the round fusiform gall, this trend is similar to the capsule of *A*. *thaliana* which became round when the *ERECTA* was knocked out [34]. We suppose the morphology of fusiform galls may fall under similar regulation to *ERECTA*. The *CUC* family also plays a role in leaf shape development [27]. We suggest that low expression levels result in the fusion of serrations or horns; therefore, leaflets and fusiform galls will show fewer serrations and horns, respectively. We suggest that the target genes perform other unknown functions in rapidly growing fusiform galls (third stage). In horned galls, all target genes were detected from the first to third stage; *CUC-2* showed the highest expression level, and the genes were not expressed at the fourth and fifth stage, that maybe because the shape of horned galls was formed during the rapid growth stage. Regression analysis showed that *CUC-2* made the greatest contribution to the formation of horns and serrations. Notably, the target genes regulated serration development on leaflets, but in galls, the serrations presented as irregular horns. In some studies, galls on leaves may also be regarded as organs of host plants [45, 46], hence the effect of host plants on galls may be considered important. In this study, we found the horns on the horned gall under regulating of the genes which regulate the serration development on the host plant leaflets.

The distribution of trichomes showed positional dependence, with the surface of *R*. *chinensis* leaflets and horned galls completely covered with trichomes, while the surface of *R*. *potaninii* leaflets and fusiform galls was only covered at the first stage. *R*. *potaninii* leaflets had the least tomentum, and the fusiform galls located on them had significantly less tomentum than horned galls. Horned galls and their host leaflets had more trichomes throughout their life cycles than fusiform galls and their host leaflets. Interestingly, galls possess more trichomes than their host; this may be due to the special ecological function of galls, which is to protect themselves from herbivorous insects (small insects have difficulty walking on or biting gall directly), thereby protecting the galling insects [47–49].

The developmental patterns of galls and their host plant leaflets showed similar traits. Even though the life cycle of *R*. *chinensis* and *R*. *potaninii* leaflets was 47 days, the *R*. *chinensis* leaflets required a longer rapid growth stage than the *R*. *potaninii* leaflets. This trend affected the galls as the horned galls also had a long rapid growth period (42 days). Furthermore, regarding changes based on volume, the maximum fold change of *R*. *potaninii* leaflets and fusiform galls was 17.4 and 7.5 times, respectively; however, the maximum fold change of *R*. *chinensis* leaflets and horned galls was 10 and 3.2 times, respectively. Therefore, *R*. *potaninii* leaflets and fusiform galls grew faster than *R*. *chinensis* leaflets and horned galls. *SPB-13* and *SPB-16* may be involved in regulating the development period of galls and leaflets, as they had high expression levels in horned galls and *R*. *chinensis* leaflets.

Fusiform galls have a shorter growth time but are larger than horned galls, and this corresponds to the cellular level, in terms of both the larger cell size and more parenchymal cells. In fusiform galls, the quantity of the cell layer increased from the first to the fourth stage and the area of cells increased throughout the developmental stage. This indicates that the enlargement of fusiform galls is accomplished by an increase in both cell number and cell size from the first to the fourth stage, but is associated with increased cell size from the fourth to the fifth stage. A small difference was found in horned galls, in which the cell area showed a limited increase from the fourth to the fifth stage, but the number of cell layers always increased throughout development. Regardless of the developmental period, the quantity of cell layer was always higher in fusiform galls than horned galls, and the cell size was always larger in fusiform galls than horned galls. Notably, the size of upper epidermal cells was larger on *R*. *potaninii* leaflets than on *R*. *chinensis* leaflets, thus it is likely that the cell size of galls is related to the cell size of the host plant.

Galls are wonderful products of interactions between inducers and host plants, and their formation involves a complex regulatory mechanism. It is commonly known that the galling process is based on a very close relationship between the insect inducer activity and the plant response, and the inducer is the trigger of gall formation. However, there is also a significant role for the plants in gall formation, because the material and gene regulation of galls are derived from plants.

## Supporting information

S1 Fig Surface of *R*. *potaninii* leaflets, fusiform galls, *R*. *chinensis* leaflets, and horned galls. (A-C) Surface of *R*. *potaninii* leaflets in the first, third, and fifth stage, respectively. (D-F) Surface of fusiform galls in first, third, and fifth stage, respectively. (G-I) Surface of *R*. *chinensis* leaflets in first, third, and fifth stage, respectively. (J-L) Surface of horned galls in first, third, and fifth stage respectively. Number of trichome decreased with development in all samples, but fusiform galls and their host plant (*R*. *potaninii* leaflets) had relatively fewer trichomes.
(TIF)

S1 Table The used primers in this study. (PDF)

## References

[1] Johansen DA. Plant Microtechnique. (Scientific Books: Plant Microtechnique). science. 1940; 120–133.

[2] Tooker JF, Helms AM. Phytohormone Dynamics Associated with Gall Insects, and their Potential Role in the Evolution of the Gall-Inducing Habit. Journal of Chemical Ecology. 2014; 40(7):742–753. doi: 10.1007/s10886-014-0457-6 25027764

[3] Graham NS and Karsten S. The adaptive significance of insect gall morphology. Trends in Ecology & Evolution. 2003; 18:512–522. doi: 10.1016/S0169-5347(03)00247-7

[4] Yang ZX, Chen XM, Foottit RG. The effect of the gall-forming aphid Schlechtendalia chinensis (Hemiptera: Aphididae) on leaf wing ontogenesis in Rhus chinensis (Sapindales: Anacardiaceae). Entomological Society of America. 2014; 107(1):242–250.

[5] Álvarez R, Martinez JJI, Mu-Oz-Viveros AL, Molist P, Abad-González J, Nafría JN, et al. Contribution of gall microscopic structure to taxonomy of gallicolous aphids on Pistacia. Plant Biology. 2016;18:868–875. doi: 10.1111/plb.12475 27259077

[6] Álvarez R, González-Sierra S, Candelas A, Martinez J. Histological study of galls induced by aphids on leaves of Ulmus minor: Tetraneura ulmi induces globose galls and Eriosoma ulmi induces pseudogalls. Arthropod-Plant Interactions. 2013; 7(6):643–650. doi: 10.1007/s11829-013-9278-8

[7] Schultz JC, Edger PP, Body MJ, Appel HM. A galling insect activates plant reproductive programs during gall development. Scientific Reports. 2019; 9:1883. doi: 10.1101/383851 30755671 PMC6372598

[8] Davey MR, Curtis IS, Gartland KMA. Agrobacterium-induced crown gall and hairy root diseases: their biology and application to plant genetic engineering. New York: Oxford University Press; 1994; 95–120.

[9] Takeda S, Hirano T, Ohshima I, Sato MH. Recent progress regarding the molecular aspects of insect gall formation. International Journal of Molecular Sciences. 2021; 22(17):9424. doi: 10.3390/ijms22179424 34502330 PMC8430891

[10] Cambier S, Ginis O, Moreau SJ, Gayral P, Hearn J, Stone GN, et al. Gall wasp transcriptomes unravel potential effectors involved in molecular dialogues with oak and rose. Frontiers in physiology. 2019; 10:926. doi: 10.3389/fphys.2019.00926 31396099 PMC6667641

[11] Favery B, Dubreuil G, Chen MS, Giron D, Abad P. Gall-Inducing Parasites: Convergent and Conserved Strategies of Plant Manipulation by Insects and Nematodes. Annual Review of Phytopathology. 2020; 58(1):1–22. doi: 10.1146/annurev-phyto-010820-012722 32853101

[12] Burks BD. The Ecology of Plant Galls. Mycologia. 1965;57(1):145.

[13] Price PW, Fernandes GW, Waring GL. Adaptive Nature of Insect Galls. Environmental Entomology. 1987; 16(1):15–24. doi: 10.1093/ee/16.1.15

[14] Kutsukake M, Meng XY, Katayama N, Nikoh N, Shibao H, et al. An insect-induced novel plant phenotype for sustaining social life in a closed system. Nature Communication. 2012; 3:1187. doi: 10.1038/ncomms2187 23149732 PMC3514493

[15] Larson KC, Whitham TG. Manipulation of food resources by a gall-forming aphid: the physiology of sink-source interactions. Oecologia. 1991; 88:15–21. doi: 10.1007/BF00328398 28312726

[16] Wool D. Galling aphids: specialization, biological complexity, and variation. Annual Review of Entomology. 2004; 49(1):175. doi: 10.1146/annurev.ento.49.061802.123236 14651461

[17] Inbar M, Wink M, Wool D. The evolution of host plant manipulation by insects: molecular and ecological evidence from gall-forming aphids on Pistacia. Molecular Phylogenetics & Evolution. 2004; 32(2):504–511. doi: 10.1016/j.ympev.2004.01.006 15223033

[18] Patankar R, Thomas SC, Smith SM. A gall-inducing arthropod drives declines in canopy tree photosynthesis. Oecologia. 2011; 167(3):701–709. doi: 10.1007/s00442-011-2019-8 21618011

[19] Ribeiro SP, Basset Y. Gall-forming and free-feeding herbivory along vertical gradients in a lowland tropical rainforest: the importance of leaf sclerophylly. Ecography. 2007; 30(5):663–672. doi: 10.1111/j.2007.0906-7590.05083.x

[20] Khan HAA, Akram W, Khan T, Arshad M, Hafeez F. Correlation of biochemical leaf traits and gall formation in six cultivars of Mango, Mangifera indica L. Pakistan Journal of Agricultural Sciences. 2017; 54(1):91–96.

[21] Martinez JJI, Moreno-González V, Jonas-Levi A, Álvarez R. Quantitative differences detected in the histology of galls induced by the same aphid species in different varieties of the same host. Plant Biology. 2018; 20:516–524. doi: 10.1111/plb.12705 29424091

[22] Ferreira BG, Oliveira DC, Moreira A, Faria AP, Guedes LM, França M, et al. Antioxidant metabolism in galls due to the extended phenotypes of the associated organisms. PLoS ONE. 2018; 13(10):e0205364. doi: 10.1371/journal.pone.0205364 30346955 PMC6197632

[23] Shorthouse JD, Wool D, Raman A. Gall-inducing insects—nature’s most sophisticated herbivores. Basic and Applied Ecology. 2005; 6:407–411. doi: 10.1016/j.baae.2005.07.001

[24] Dengler NG, Tsukaya H. Leaf Morphogenesis in Dicotyledons: Current Issues. International Journal of Plant Sciences. 2001; 162(3):459–464. doi: 10.1086/320145

[25] Du F, Guan C, Jiao Y. Molecular Mechanisms of Leaf Morphogenesis. Mol Plant. 2018; 11(9):1117–1134. Epub 2018/07/01. doi: 10.1016/j.molp.2018.06.006 . 29960106

[26] Vroemen CW. The CUP-SHAPED COTYLEDON3 Gene Is Required for Boundary and Shoot Meristem Formation in Arabidopsis. Plant Cell Online. 2003; 15:563–577. doi: 10.1105/tpc.012203 12837947 PMC165401

[27] I Tetsuya, A Mitsuhiro, T Shinobu, T Masao. Involvement of CUP-SHAPED COTYLEDON genes in gynoecium and ovule development in Arabidopsis thaliana. Plant & Cell Physiology. 2000; 41(1):60–67. doi: 10.1093/pcp/41.1.60 10750709

[28] Hasson A, Plessis A, Blein T, Adroher B, Grigg S, Tsiantis M, et al. Evolution and Diverse Roles of the CUP-SHAPED COTYLEDON Genes in Arabidopsis Leaf Development. Plant Cell. 2011; 23(1):54–68. doi: 10.1105/tpc.110.081448 21258003 PMC3051246

[29] Kawamura E, Horiguchi G, Tsukaya H. Mechanisms of leaf tooth formation in Arabidopsis. Plant Journal. 2010; 62(3):429–441. doi: 10.1111/j.1365-313X.2010.04156.x . 20128880

[30] Aida M, Ishida T, Fukaki H, Fujisawa H, Tasaka M. Genes involved in organ separation in Arabidopsis: an analysis of the cup-shaped cotyledon mutant. Plant Cell. 1997; 9(6):841–857. doi: 10.1105/tpc.9.6.841 9212461 PMC156962

[31] Takada S, Hibara KI, Ishida T, Tasaka M. The CUP-SHAPED COTYLEDON1 gene of Arabidopsis regulates shoot apical meristem formation. Development. 2001; 128(7):1127–1135. doi: 10.1242/dev.128.7.1127 11245578

[32] Hui Z, Xiaoyu L, Müller-Xing R, Xingshun S, Qian X. Research Progress of ERECTA in Arabidopsis. Chinese Agricultural Science Bulletin. 2018; 34(15):93–99.

[33] Shpak ED. Diverse Roles of ERECTA Family Genes in Plant Development. Journal of Integrative Plant Biology. 2013; 55(12):1238–1250. doi: 10.1111/jipb.12108 24016315

[34] Tisne S, Reymond M, Vile D, Fabre J, Dauzat M, Koornneef M, et al. Combined Genetic and Modeling Approaches Reveal That Epidermal Cell Area and Number in Leaves Are Controlled by Leaf and Plant Developmental Processes in Arabidopsis. Plant Physiology. 2008; 148(2):1117–1127. doi: 10.1104/pp.108.124271 18701672 PMC2556812

[35] Chen M-K, Wilson RL, Palme K, Ditengou FA, Shpak ED. ERECTA Family Genes Regulate Auxin Transport in the Shoot Apical Meristem and Forming Leaf Primordia Plant Physiology. 2013; 162(4):1978–1991. doi: 10.1104/pp.113.218198 23821653 PMC3729776

[36] Kyozuka Junko. Chapter Seven—Grass Inflorescence: Basic Structure and Diversity. Advances in Botanical Research. 2014; 72:191–219. doi: 10.1016/B978-0-12-417162-6.00007-9

[37] Xu M, Hu T, Zhao J, Mee-Yeon P, Earley KW, Wu G, et al. Developmental Functions of miR156-Regulated SQUAMOSA PROMOTER BINDING PROTEIN-LIKE (SPL) Genes in Arabidopsis thaliana. PLoS Genetics. 2016; 12(8):e1006263. doi: 10.1371/journal.pgen.1006263 27541584 PMC4991793

[38] Chen X, Zhang Z, Liu D, Zhang K, Li A, Mao L. SQUAMOSA Promoter-Binding Protein-Like transcription factors: Star players for plant growth and development. Journal of Integrative Plant Biology. 2010; 52(11):946–951. doi: 10.1111/j.1744-7909.2010.00987.x 20977652

[39] Ye BB, Zhang K, Wang JW. The role of miRi56 in rejuvenation in Arabidopsis thaliana. Journal of Intergrative Plant Biology. 2020; 62(5):550–555. doi: 10.1111/jipb.12855 31305005

[40] Yang ZX, Chen XM, Havill NP, Feng Y, Chen H. Phylogeny of Rhus gall aphids (Hemiptera: Pemphigidae) based on combined molecular analysis of nuclear EF1α and mitochondrial COII genes. Blackwell Publishing Asia. 2010; 13(3):351–357. doi: 10.1111/j.1479-8298.2010.00391.x

[41] Chen X, Feng Y. An introduction to resource entomology. Beijing: Science Press; 2009; 45–56.

[42] Lu Q, Chen H, Wang C, Yang ZX, Lü P, Chen MS, et al. Macro- and Microscopic Analyses of Anatomical Structures of Chinese Gallnuts and Their Functional Adaptation. Scientific Reports. 2019; 9(1):5193. doi: 10.1038/s41598-019-41656-6 30914739 PMC6435719

[43] Lu Q, Chen X, Yang Z, Bashir NH, Chen H. Molecular and Histologic Adaptation of Horned Gall Induced by the Aphid Schlechtendalia chinensis (Pemphigidae). International Journal of Molecular Sciences. 2021; 22(10):5166. doi: 10.3390/ijms22105166 34068250 PMC8153119

[44] Lu Q, Bashir NH, Wu H-X, Wang W, Zhang J, Cui Y, et al. Structure, Distribution, Chemical Composition, and Gene Expression Pattern of Glandular Trichomes on the Leaves of Rhus potaninii Maxim. International Journal of Molecular Sciences. 2021; 22(14):7312. doi: 10.3390/ijms22147312 34298931 PMC8303848

[45] Ferreira BG, Álvarez R, Bragança GP, Alvarenga DR, Pérez-Hidalgo N, Isaias R. Feeding and other gall facets: patterns and determinants in gall structure. The Botanical Review. 2019; 85(1):78–106. doi: 10.1007/s12229-019-09207-w

[46] Takei M, Yoshida S, Kawai T, Hasegawa M, Suzuki Y. Adaptive significance of gall formation for a gall-inducing aphids on Japanese elm trees. Journal of Insect Physiology. 2015; 72:43–51. doi: 10.1016/j.jinsphys.2014.11.006 25437243

[47] Nyman T, Widmer A, Roininen H. Evolution of gall morphology and host-plant relationships in willow-feeding sawflies (Hymenoptera: Tenthredinidae). Evolution. 2000; 54(2):526–533. doi: 10.1111/j.0014-3820.2000.tb00055.x 10937229

[48] Dixon KA, Lerma RR, Craig TP, Hughes KA. Gall Morphology and Community Composition in Asphondylia flocossa (Cecidomyiidae) Galls on Atriplex polycarpa (Chenopodiaceae). Environmental Entomology. 1998; (3):592–599. doi: 10.1093/ee/27.3.592

[49] Álvarez R, Encina A, Hidalgo NP. Histological aspects of three Pistacia terebinthus galls induced by three different aphids: Paracletus cimiciformis, Forda marginata and Forda formicaria. Plant Science. 2009; 176(2):303–314. doi: 10.1016/j.plantsci.2008.11.006

