# Supplementary figures and images for: A study on the effect of host plants on Chinese gallnut morphogenesis
Source: PLoS One. 2023 Mar 22;18(3):e0283464. doi: 10.1371/journal.pone.0283464 (PMC10032517; doi:10.1371/journal.pone.0283464)

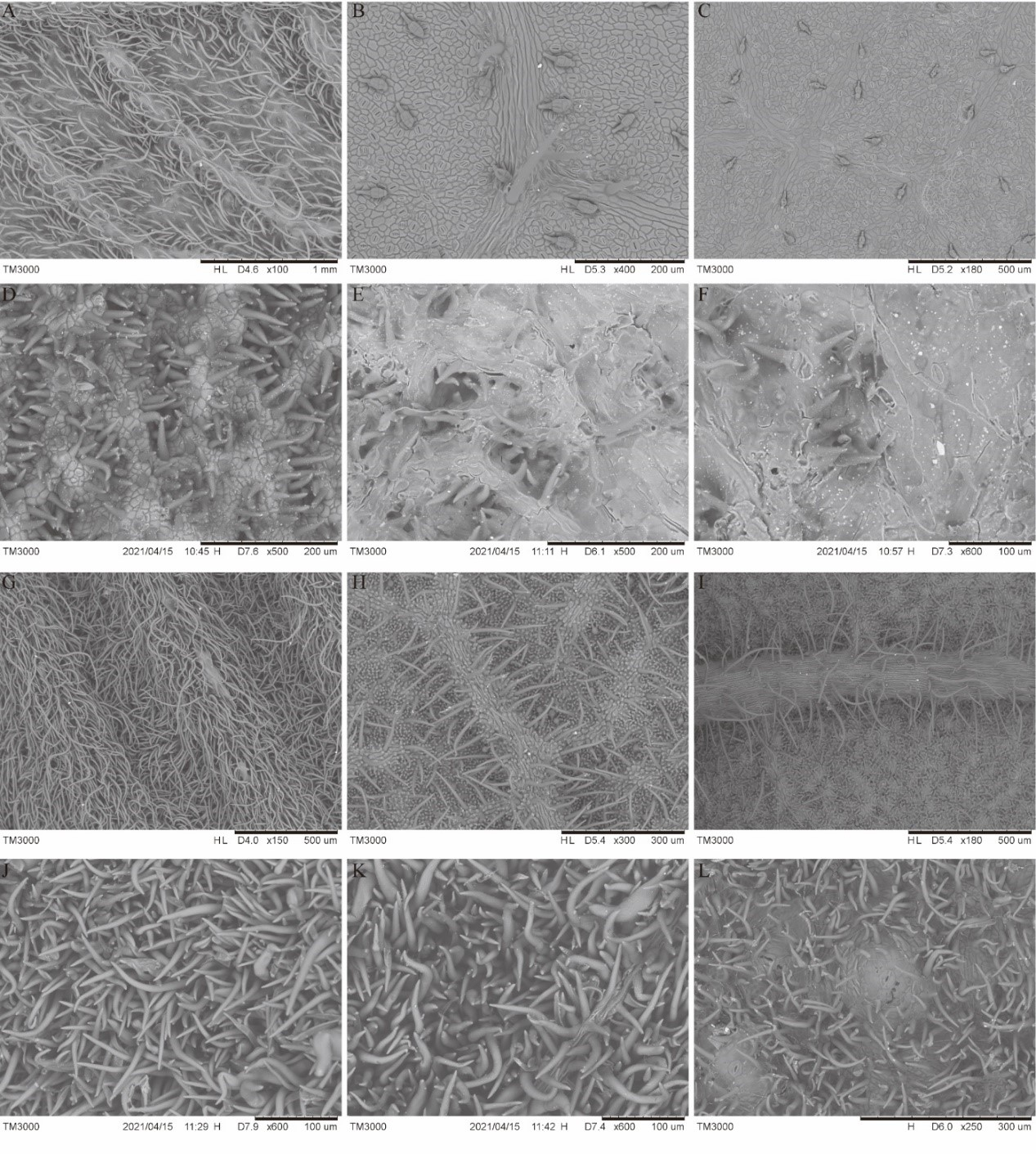

Supplement: S1 Fig — (A-C) Surface of R. potaninii leaflets in the first, third, and fifth stage, respectively. (D-F) Surface of fusiform galls in first, third, and fifth stage, respectively. (G-I) Surface of R. chinensis leaflets in first, third, and fifth stage, respectively. (J-L) Surface of horned galls in first, third, and fifth stage respectively. Number of trichome decreased with development in all samples, but fusiform galls and their host plant (R. potaninii leaflets) had relatively fewer trichomes. (TIF) [file pone.0283464.s001.tif]
