# Supplementary material for: A study on the effect of host plants on Chinese gallnut morphogenesis
Source: PLoS One. 2023 Mar 22;18(3):e0283464. doi: 10.1371/journal.pone.0283464 (PMC10032517; doi:10.1371/journal.pone.0283464)
Supplement: S1 Table — (PDF) [file pone.0283464.s002.pdf]

S1 Table. The used primers in this study.

	Target genes	primers
R. chinensis leaf and horned gall	CUP-2-sense	TGCTCTTCTGAAACACCCTTGA
	CUP-2-anti-sense	CCAACTGGGCTGAGAACTAACA
	CUP-3-sense	GGTTTAGATTCCACCCTACTGATG
	CUP-3-anti-sense	CTCCCGTTGCTCTGTTTGTTC
	ERECTA-1-sense	GGTCACAGTCACCATCAGCGA
	ERECTA-1-anti-sense	CTCCCAATCTCCTCCACAGC
	ERECTA-2-sense	CTCAGGAAGTCGTAATGGCACA
	ERECTA-2-anti-sense	CTCTTGCGTGCCTTGATGTTC
	SPB-1-sense	GTTGGCATTACTACTGCTGTCCG
	SPB-1-anti-sense	CAATCCCTCCTTCTCAGTCCTT
	SPB-2-sense	TCAACCTGCCCACAGACCAT
	SPB-2-anti-sense	CAACGAACGACGAAGGAAGAAT
	tublin-sense	GGAGGAGGTGACGATGCTTTC
	tublin-anti-sense	TGTTGGCGGCATCTTCCTT
R. potaninii leaf and fusiform gall	CUP-2-sense	TGACTTCGGAGTTAGTTGGGATG
	CUP-2-anti-sense	TGATAGTTCTCGGCTGCTGCTT
	CUP-3-sense	ACCACCAACAGCAACAACGATA
	CUP-3-anti-sense	TCCATTCTTTGCCCTCCTACTT
	ERECTA-1-sense	ACAAAGCACCACCACATCGTCT
	ERECTA-1-anti-sense	TGGTTTCGGTGTCTTAGTTCTGG
	ERECTA-2-sense	TGAGAGGAGGCTAAATGAAGGG
	ERECTA-2-anti-sense	GACTGCCCTTGAAACAAACTCC
	SPB-1-sense	CCTTCTACCACCATCAACACCC
	SPB-1-anti-sense	CCACCTAAGTTCAACCCAATCC
	SPB-2-sense	GTAATGCTTAGCGTGGGTCAGA
	SPB-2-anti-sense	GGATTGGGTTGAACTTAGGTGG
	tublin-sense	GGCAGCAGAATGAAGCGGT
	tublin-anti-sense	ACAGCAGTTTCCCTATCCCTCA
